# Improving access to tuberculosis preventive treatment for children in Ethiopia: designing a home-based contact management intervention for the CHIP-TB trial through formative research

**DOI:** 10.1186/s12913-024-11451-9

**Published:** 2024-09-10

**Authors:** Nicole Salazar-Austin, Alanna J. Bergman, Christiaan Mulder, Carrie Tudor, Fiseha Mulatu, Gidea Conradie, Richard E. Chaisson, Jonathan E. Golub, Gavin Churchyard, Ahmed Bedru, Deanna Kerrigan

**Affiliations:** 1 Department of Pediatrics, Johns Hopkins University School of Medicine, 200 N. Wolfe Street Room 3147, Baltimore, MD 21287 USA; 2 Johns Hopkins Center for Infectious Disease and Nursing Innovation, Baltimore, MD USA; 3 Department of TB Elimination and Health System Innovations, KNCV Tuberculosis Foundation, The Hague, The Netherlands; 4 Amsterdam Institute for Global Health and Development, Amsterdam University Medical Centres, Amsterdam, The Netherlands; 5 KNCV Ethiopia, Addis Ababa, Ethiopia; 6 The Aurum Institute, Johannesburg, South Africa; 7 Department of Medicine, Johns Hopkins University School of Medicine, Baltimore, MD USA; 8 Department of Prevention and Community Health, George Washington University Milken Institute School of Public Health, Washington D.C., USA

**Keywords:** Tuberculosis prevention, Home-based, Community health worker

## Abstract

**Background:**

Tuberculosis (TB) preventive treatment (TPT) is a long-standing recommendation for children exposed to TB but remains poorly implemented. Home-based contact management may increase access and coverage of TPT among children exposed to TB in their households.

**Methods:**

Sixty in-depth interviews were conducted with key informants including program managers, TB providers (known as TB focal persons), health extension workers and caregivers whose children had recently engaged with TB prevention services in Oromia, Ethiopia in 2021 to understand the barriers and facilitators to providing home-based TB prevention services for children aged < 15 years. Thematic content analysis was conducted including systematically coding each interview.

**Results:**

Home-based services were considered a family-centered intervention, addressing the time and financial constraints of clients. Stakeholders proposed a task-shared intervention between health extension workers and facility-based TB focal persons. They recommended that TB services be integrated into other home-based services, including HIV, nutrition, and vaccination services to reduce workload on the already overstretched health extension workers. Community awareness was considered essential to improve acceptability of home-based services and TPT in general among community members.

**Conclusions:**

Decentralization of TPT should be supported by task-sharing initiation and follow up between health extension workers and facility-based TB focal persons and integration of home-based services. Active community engagement through several existing mechanisms can help improve acceptability for both home-based interventions and TPT promotion overall for children.

**Trial registration:**

The results presented here were from formative research related to the CHIP-TB Trial (Identifier NCT04369326) registered on April 30, 2020. This qualitative study was separately registered at NCT04494516 on 27 July 2020.

**Supplementary Information:**

The online version contains supplementary material available at 10.1186/s12913-024-11451-9.

## Background

Globally, over one million children develop tuberculosis (TB) disease each year resulting in over 200,000 child deaths [1]. TB preventive treatment (TPT) is highly effective at preventing progression from TB infection to TB disease but remains poorly implemented. Only 55% of household contacts aged under five years and 10% of household contacts aged five or older years were started on TPT between 2018 and 2022 [1]. Studies show nearly two-thirds of child contacts aged less than five years are either not identified or not linked to facility-based care [2]. Community- or home-based care has expanded access and improved treatment outcomes for TB and HIV treatment and prevention [3–7]. Bringing contact investigation and TPT services to the home could improve identification of close child contacts, increase TPT initiation and TPT completion [6, 8].

Ethiopia is a high TB and TB/HIV burden country with a TB incidence rate of 126 per 100,000 persons [1]. Ethiopia was an early adopter of short-course TPT and have included both three months of weekly rifapentine and isoniazid (3HP) and three months of daily rifampicin and isoniazid (3RH) in the guidelines since 2018 [9]. The Ethiopian health care system is comprised of three tiers, the primary health care units [health post, health center and primary hospital], general hospitals and specialized hospitals that deliver primary, secondary and tertiary care to the people of Ethiopia. The primary health care units are supported by a strong community-based health extension program [10]. The program aims to enhance access to health promotion, disease prevention and selective treatment services. Since its inception in 2003, the program has established health posts in each village (Kebele) that utilize community health workers or health extension workers (HEWs), to provide services to 3,000–5,000 persons in the community. These HEWs receive up to two years of dedicated pre-service training followed by ongoing supportive supervision from healthcare workers from an affiliated health center. HEWs are paid to provide 18 packages of care that are delivered at either the home or the health post. They receive monthly supportive supervision by staff from their affiliated health centers. HEWs provide pediatric treatment for malaria, pneumonia, neonatal sepsis, diarrhea with dehydration, and malnutrition [11–14]. They also conduct TB screening and recently started providing directly observed therapy for people undergoing TB treatment. The health extension program has been critical to expanding access to health care services in rural, pastoral and urban communities and in improving health outcomes, including a two-thirds reduction in under-5 mortality since 2003. A comprehensive evaluation of the program was conducted in 2020 [10, 15]. Existing data suggest that HEWs sometimes lack the knowledge and supplies needed for service delivery. Limited awareness of available services and perhaps limited trust in the HEWs’ skills may lead to underutilization of HEW services at the home and health post level [10]. Additionally, motivation and commitment are noted as challenges that are being addressed by balancing the volume of HEWs’ work and providing incentives.

CHIP-TB is a cluster randomized trial assessing home-based versus facility-based contact management in 18 health centers in the East Shewa Zone of Oromia, Ethiopia (NCT04369326). During a formative phase of the project, we conducted qualitative, semi-structured in-depth interviews with program managers, TB providers, HEWs and caregivers of children exposed to TB to explore the feasibility and acceptability of providing home-based contact management to inform optimal implementation within the CHIP-TB Trial.

## Methods

This formative qualitative research was conducted prior to the initiation of the CHIP-TB trial [8]. Interviews were conducted from June to October 2021. The study utilized a cross-sectional design and enrolled 60 stakeholders who participated in one individual, in-depth interview.

### Study setting

At the time of data collection, updates to the Ethiopian National TB Guidelines had extended the recommendation of TPT for close child contacts < 5 years of age to those < 15 years of age. HEWs were tasked with visiting households of persons being treated for TB to provide directly observed therapy; but most persons being treated with TB were still receiving facility-based directly observed therapy daily. Contact tracing was tasked to HEWs, but was variably implemented; a minority of health centers sent HEWs to households to enumerate household contacts. TB evaluation, TPT initiation and TPT follow up (together known as contact investigation) all occurred in the health center by a TB provider known in Ethiopia as a TB focal person.

### Study population

We purposively recruited 15 program managers, 15 TB providers including TB focal persons and pediatricians, and 15 HEWs. Participants had to be a TB, MCH or other relevant program manager or policy maker, a TB provider or an HEW from one of the Ethiopian clinics in which the subsequent trial would enroll from or an adult caregiver, at least 18 years old, of a child < 15 years old who was exposed to TB in the last two years who was evaluated and offered either TPT or TB treatment. We attempted to vary gender, age and health center to capture the widest range of perspectives possible. This was achieved through reiterative review of all enrolled participants on a weekly basis. The program managers included TB/HIV, Maternal and Child Health (MCH), and Health Extension Program coordinators at the local and regional levels of who served at the East Shewa Zone of Oromia. Populations represented in this study include rural and semi-urban populations, but not pastoral or urban populations. Additionally, we recruited 15 caregivers of children who were investigated for TB as part of a household contact investigation in the previous two years. Caregivers were selected based on their experience and willingness to narrate their experience with TB preventive services and TPT. We attempted to purposively select caregivers with varied perspectives and experience including those with and without children 5–15 years of age, those whose children did and did not screen TB positive, those who did and did not accept TPT for their children, and those who were and were not the person being treated for TB themselves. Because the Ethiopian Ministry of Health had recently increased the age for TPT from < 5 years to < 15 years, no caregivers had experience providing TPT to children 5 to < 15 years of age, however, we purposively selected caregivers with children this age to ensure challenges in this age group could be considered. TB providers, HEWs and caregivers were all recruited from the health centers included in the CHIP-TB trial and their associated primary hospitals. All health centers provided TB services; none provided TPT initiation at the home or the health post.

### Procedures

A semi-structured topical field guide (Supplementary files 1 and 2) was developed to explore potential barriers, facilitators, and benefits of home-based contact investigation and TPT services performed by HEWs, organizational assumptions and attitudes by and about community health workers and best practices, anticipated challenges, and solutions to program implementation. Interviewers received training on each question, it’s overall purpose and the type of information we were attempting to understand. The tool was pilot tested among the interviewers themselves and among NGO/collaborator program staff.

All interviews were approximately one-hour and conducted one-on-one in Amharic or Afaan Oromo by male research assistants who received training in qualitative research and interviewing techniques. Interviewers were not involved in the parent CHIP-TB trial and had no prior relationship with the participants. Training was conducted on social desirability bias. The interview began with a statement explaining the purpose of the interview, the desire to understand the participant’s views, and that there were no right or wrong answers to our questions.

Interviews were paused after the initial 10 interviews and again after a second set of 10 interviews. At this time, interviewing skills, transcription and translation were assessed to ensure high quality data collection, transcription and translation. Interviews then continued until 15 participants in each stakeholder group were enrolled. Thematic redundancy was achieved in each stakeholder group. Adequate redundancy was identified when the analytic team noted a dearth of emergent codes indicating a low likelihood of new themes or information to be gained from additional interviews.

### Analysis

All interviews were audio-recorded, transcribed, and translated. All translators had advanced degrees and were fluent in either Afaan Oromo and English or Amharic and English. Transcripts were analyzed using thematic content analysis with both a deductive and inductive approach for both a priori and emergent domains of interest. A priori codes were developed based on the research question and interview guides (e.g., health system structure, health system access, HEW roles and responsibilities, perception of intervention, and intervention strategy). Emergent codes were created in response to identification and exploration of unexpected domains during the coding process (e.g., perceived need for TPT and TB/TPT awareness). The coding process was performed in parallel by three analysts who all had knowledge of the study and settings and in concert with the study’s primary investigator. The three analysts made iterative revisions to the codebook throughout the coding process. Memo-ing was used to record analysts’ observations and reflections of the data, coding process and theme development. Coders met weekly to discuss emergent codes and preliminary themes to increase consistency in coding, to discuss reflexive interpretations of the data and to consider divergent viewpoints. This approach allowed for anticipated and unexpected themes to emerge. Local Ethiopian research staff provided clarity and context for the data, verifying preliminary themes when needed. Codes were organized and stored using ATLAS.ti© analytical software [16]. Codes were assessed and analyzed within and across each stakeholder group. Code relationships and themes were identified through consensus and summarized using a template. The themes were continuously reviewed and discussed by the research team. Table 1 provides examples of codes that informed the generation of each study theme. Illustrative quotes highlight participant views around each theme. Quotes are described using gender, role, and years of work experience to show the breadth of participant views. Finally, a figure was developed to summarize the underlying context provided in the background along with a summary of the participants’ descriptions of the barriers, facilitators and suggested implementation strategies for a home-based contact investigation intervention.

**Table 1:** Main study themes and examples of codes per thematic area

Study Themes and Subthemes	Code	Code Definitions
Theme 1: Home-based TB preventive services overcome family-level barriers	Perception of Intervention	Stakeholder feelings or perceptions about the intervention
	Health system access	Structural and cultural factors that impact access to health facilities or access to health services
	Caregiver Expenses/cost	Expenses incurred or lost by family/patients/caregivers as a result of TB contact investigation including travel to health center or health post for screening/diagnosis/DOT, food, lost work/income, etc
	Caregiver Transportation	Challenges related to patient transport or travel needs to access TB preventive care
	Caregiver Time	Time spent traveling to health center/post/hospital for contact investigation and TB preventive services
	^#^Caregiver centered care	Services and adaptations to care that reflect the needs and preferences of both children and their caregivers and/or examples of how current programming is not caregiver-centered
	Perception of caregiver behavior	Stakeholder feelings or perceptions about the caregiver behavior
Theme 2: Home-based TB prevention services will improve access to care and TPT coverage	Challenges to contact tracing	Challenges related to contact tracing
	Challenges to contact investigation	Challenges related to screening for symptoms or screening contacts of TB clients
	Perception of Intervention	Stakeholder feelings or perceptions about the proposed intervention
	^#^Awareness	Community or individual awareness about TB or TPT, or the need to increase that awareness
	Caregiver motivation	Describes caregiver motivation and/or how to motivate caregivers and children to stay on TPT
	^#^Community benefit	Discussion of TPT's or the intervention's public health benefits to the community (rather than individual outcomes)
Theme 3: Acceptability and Feasibility of HEW-initiated TPT	Perception of intervention	Stakeholder’s feelings or perceptions about the intervention
	Intervention Strategy	Stakeholder’s suggestions about how intervention could or should work
	Perception of HEW	Stakeholder’s feelings or perceptions about HEW services, attitudes, etc
	HEW roles and responsibilities	Perceived roles and responsibilities of HEWs
	Provider and HEW collaboration	How providers and HEWs work together to care for people with TB infection or disease and how they identify those at risk
	Supervision	Supervision including supportive supervision of HEWs by providers/managers or HEWs supervising community groups
	Training	Training needs of healthcare providers and HEWs
	Feedback	Feedback from community regarding HEWs
	^#^Experience sharing	Experience sharing as a way of training or sharing information / practices between providers and HEWs
	HEW Motivation	HEWs’ motivation to complete house-to-house work or what is needed to motivate HEWs to do their work
	Tools/ job aids	Provider and HEWs needs / requests for job aids, guidelines, other tools, etc
Theme 4: Overworked, understaffed health extension program and the need for service integration	Perception of intervention	Stakeholder’s feelings or perceptions about the proposed intervention
	TPT challenge	Challenges and barriers to TPT implementation (exclusive to proposed intervention)
	Staffing	Challenges related to staffing levels at either health facility or health post
	^#^Workload	Challenges related to how much work or duties a healthcare worker has
	Integrated services	Integrating TPT services with other HEW services (e.g., sanitation, vaccination, nutrition etc.) including integration into the HEWs’ home-based packages of care
Theme 5: Community awareness as a facilitator of TPT and of the home-based intervention:		
*Theme 5a) Harnessing community awareness to improve community-level acceptability of home-based TPT*	^#^TB/TPT Awareness	Community or individual awareness about TB or TPT, or the need to increase that awareness
	^#^Community engagement	Need for collaboration or engagement with community members or community networks
	Health education: IEC materials	Need for posters, media, pamphlets, etc. to use for health education on TPT in the community
*Theme 5b) Community awareness to improve TPT uptake among skeptical caregivers who view their well children as not needing medication*	^#^TB/TPT Awareness	Community or individual awareness about TB or TPT, or the need to increase that awareness
	^#^Perceived need for TPT	If a child is "not sick" (asymptomatic TB infection) there is no need to take medication, especially when the medicine has side effects
*Theme 5c) Community awareness to reduce stigma as a barrier to intervention acceptability*	^#^TB/TPT Awareness	Community or individual awareness about TB or TPT, or the need to increase that awareness
	Stigma	TB or TPT-related stigma and discrimination
	^#^Caregiver behavior/attitudes	Caregiver behaviors or attitudes towards TPT

^#^Delineates emergent theme

### Ethical considerations

Program managers, providers, and HEWs were approached directly by study staff while caregivers were recruited with the assistance of health center staff. Interviews took place in the program manager’s office, in the provider’s and HEW’s associated health center or health post, and in the caregiver’s home or associated health center, according to the participants’ preference. No other staff or family members were present.

All participants received oral and written information about the study’s purpose and the risks and benefits of participation. Voluntary participation was stressed including their right to withdraw from the study at any time for any reason. Data were anonymized and stored securely to avoid identification of specific individuals. All study participants provided written informed consent prior to voluntary study participation with audio recording of the interview. Participants were remunerated for their time. This study was reviewed and approved by the Oromia Regional Health Bureau Public Emergency and Health Research Directorate Institutional Review Board (BEFO/HBTFH/ 460/23/03/2012), the Johns Hopkins Medicine Institutional Review Board (IRB00237243), and the World Health Organization’s Ethics Review Committee (ERC.0003244).

## Results

A total of 60 participants were interviewed including 15 program managers, 15 providers, 15 health extension workers and 15 caregivers (Table 2). Training backgrounds included a mix of nurses, physicians, and public health professionals. All health extension workers were female and 73% had more than 10 years of experience. Caregivers were all female with a median age of 28 years (range 20–40). No one refused to participate in the interviews.

**Table 2:** Participant Demographics

	**Program Managers** ***N* = 15**	**Providers** ***N* = 15**	**Health Extension Workers** ***N* = 15**	**Caregivers** ***N* = 15**
**Gender**				
Female	4 (27%)	13 (87%)	15 (100%)	15 (100%)
Male	11 (73%)	2 (13%)	-	-
**Training Background**				
Nurse	5 (33%)	-	-	-
Public Health	9 (60%)	-	-	-
Physician	1 (7%)	2 (13%)	-	-
TB Focal Person	-	12 (80%)	-	-
MCH	-	1 (7%)	-	-
HEW	-	-	15 (100%)	-
**Median Length of Time at Current Post**				
< 5 years	11 (73%)	11 (73%)	2 (13%)	-
5–10 years	3 (20%)	4 (27%)	2 (13%)	-
> 10 years	1 (7%)	0 (0%)	11 (73%)	-
**Household Composition**				
Houses with children < 5 years only	-	-	-	3 (20%)
Houses with children 5–14 years only	-	-	-	4 (27%)
Houses with children 0–14 years	-	-	-	8 (53%)
**Relationship to Children**				
Parent	-	-	-	15 (100%)
**Age**				
Median Age (range)	-	-	-	28 years (20, 40)

The results are presented in five themes: (1) Home-based TB preventive services overcome family-level barriers; (2) Home-based TB prevention services will improve access to care and TPT coverage; (3) Acceptability and feasibility of HEW-initiated TPT; (4) Overworked, understaffed health extension program and the need for service integration (5) Community awareness as a facilitator of TPT and of the home-based intervention. As we attempted to center children and caregivers affected by TB, the first two themes address the perception of the intervention as person-centered, with the potential to alleviate current challenges in TPT uptake and completion. The third theme discusses the acceptability and feasibility of the intervention. The final theme was based on an emergent code, *community awareness,* which was viewed as an essential component of TPT success overall, and to the success of the intervention. The final theme is divided into three subsections detailing the broad implications of community awareness as presented by stakeholders. A summary of the underlying context along with a summary of the participants’ descriptions of the barriers, facilitators and suggested implementation strategies for a home-based contact investigation intervention are summarized in Table 3.

**Table 3:** Context, Barriers, Facilitators and Implementation Strategies for a Home-based Contact Investigation Intervention Amongst Health Extension Workers in Ethiopia

**Overall Context**	• Ethiopia remains a high TB burden country and has poor TPT coverage among children • TB care and prevention services have largely been facility-based in Ethiopia • Time, cost of transportation, lost wages, poor quality roads, and long distances to health facilities are significant barriers to families seeking TPT for their children • The perception of medication as “treatment” in an otherwise healthy child is a significant barrier to uptake and completion of TPT • Ethiopia has a cadre of healthcare workers, called health extension workers, that provide home-based and community-based services that aim to enhance access to health promotion, disease prevention and selective treatment services that have significantly contributed to reducing child mortality over the last 20 years		
**Home-based contact investigation** (includes contact tracing, TB evaluation, TPT initiation and TPT follow up)	**Barriers and Facilitators**	**Implementation Strategies**	**Needed to support implementation**
	**Facilitators** • Reduces caregiver burden (time, cost, etc.) • Reduced waiting time and clinic congestion • Home-based contact investigation will increase identification of household contacts • Home-based programming elevates importance of TPT in individual households and in the community • Reduced transmission of communicable illness in the health center (TB, COVID-19 etc.)	1. **Task-sharing** contact investigation and TPT management among HEW and TB focal person 2. **Service integration** to offset increased HEW workload 3. **Community awareness** campaigns to improve family-level acceptability of intervention and TPT	• Education on TB infection & TPT for HCWs, caregivers and community • Training on intervention protocols and implementation to HEWs, TB focal persons, pharmacists, etc • Supportive supervision of HEWs to ensure fidelity to intervention and maintenance of clinical sills • HEW medical record for family folder • Clinical support tools to assist with decision making • Registration book • Referral papers • Portable scale • Personal protective equipment • Transport for HCWs • Drug forecasting and ordering
	**Barriers** • Concern that necessary equipment for adequate contact investigation will not be available in the household • HEWs lack transportation or adequate reimbursement to make home visits in rural areas • HCWs lack transportation for supervising HEWs in community • Overworked, understaffed health extension program • Perceived poor motivation and commitment of HEWs • Home-visits are potentially stigmatizing • Increased access to TPT may result in drug stock outs		

### Theme 1: Home-based TB preventive services overcome family-level barriers

All stakeholder groups described home-based TPT services as person-centered, saving children and their caregivers both time and money. Stakeholders repeatedly discussed how a home-based intervention would cut down on transportation costs to and from health centers for the multiple visits needed for TB screening, TPT initiation and TPT follow-up. “*Some residents in this area are far from the health facility, we must pay for transportation, which is hard for us. We can collect medication from the health center but distance and transportation cost a lot. Actually, it is for my health and to benefit me. Additionally, we will be happy if they offer service at the household level*” (Parent, female, TB survivor, age 40).

Physical transport was also made difficult by unpaved roads that often flood in the rainy season, the presence of multiple children of varying ages per household, and the struggle for caregivers to physically make the trip when the caregiver was suffering from TB disease. Stakeholders also acknowledged the time spent traveling to and from health centers for TPT services resulted in lost productivity and wages for the caregivers and school interruptions for children, furthering the appeal of a home-based intervention.

Despite high overall acceptability from caregivers, one caregiver lacked confidence in home-based evaluation, concerned that the tools needed to evaluate a child for TB could not be accessed in the household. “*Home-based care might be doubtful because, I do not feel comfortable giving medication without examining the status of the child, without measuring the weight of the child*” (Parent, female, age 28). This concern did not extend to the overall acceptability of TPT or HEW-initiated TPT, but rather a concern about quality assurance in the home. The same caregiver remarked, “*I want my children to be healthy and free from illness. If they bring medication to my home, I will be happy with that. If they come home, bring medication for the children my door is always open for them. I am ready to utilize the service for my children*” (Parent, female, age 28).

### Theme 2: Home-based TB prevention services will improve access to care and TPT coverage

Because the home-based intervention addressed key patient-level barriers, stakeholders felt the home-based intervention would expand access to TB prevention services for those who may not otherwise seek facility-based care. *“We can reach each household in their village. Additionally… exposed children might suffer from the illness or die without getting treatment service, which can be addressed, through a home visit. We can examine children in their homes; we can examine them by touching their bodies and observing them in front of their families… Families, having the poor motivation to visit the health facility, those who have low or no awareness about health care services will also have the chance to get health services in their household” (HEW,* > *10 years work experience, female).*

HEWs, providers, and program managers all predicted that home-based services would lead to improvements in contact identification and management of children at risk for TB disease, “*I think we can get 100% of vulnerable children if the service is implemented at the home level… There may be vulnerable children who are hidden in the home that parents do not bring to the health center. So, I think it would be better to implement this service at home to get all the vulnerable children*” (TB focal person < 5 years work experience, male).

Furthermore, integration of TB preventive services into home-based services would elevate the importance of TB prevention to community members, and may result in improved TPT outcomes, "*Especially if we give medication in their house, they feel happy because they feel how much we are concerned about their health*" (Pediatrician < 5 years work experience, male).

### Theme 3: Acceptability and feasibility of HEW-initiated TPT

Most HEWs felt qualified to implement the intervention. Although they acknowledged a need for training and supportive supervision, HEWs were motivated to address the community’s needs. *“There is nothing that can hinder us if you fulfill the required materials for us. We work for the sake of our community benefit, for our family, for our fathers and mothers*” (HEW, > 10 years work experience, female). Many HEWs saw screening, initiating, and maintaining TPT as a natural extension of their existing services and cited other HEW-led screening activities as evidence of their proficiency, “*We are not new to this type of work, we had completed several screening activities, and the only difference in the current intervention is focusing on children”* (HEW, > 10 years work experience, female). HEWs were eager to deliver care that reduced the burden of the community. HEWs physical presence in the community provides a direct view into the home and a transparency with the community that is not shared by providers and program managers. HEWs therefore felt they were the best situated to provide TPT services due to their trusted status. One HEW remarked, *“It should be health extension workers [initiating TPT] because women may tell health extension workers about all things in their families. They know HEWs and are not afraid to consult them”* (HEW, > 10 years work experience, female). This perception was verified by caregivers who viewed HEWs as trusted and skilled neighbors, “*They are doing their best to teach the community. They are giving good follow-up and give attention to our questions. They are open for consultation…they are kind and friendly to help patients*” (Parent, female, age 32).

Providers and program managers had differing opinions about who should initiate treatment. Some providers and program managers had an immediate and almost reflexive response that TPT services should be initiated by facility-based health professionals, but conceded this wasn’t feasible given the volume of their facility-based duties. A pediatrician reasoned, *“I will be happy if I got to their home every day and assist the way they are taking medication. However, if I am going to the community to offer home-based services, the activities in the health center will be affected. No one can cover the service I am offering in the health center”* (Pediatrician < 5 years work experience, male). Notably, stakeholders did not express concern about HEWs’ skills and capacity to accurately screen for TB symptoms and initiate TPT. Instead, some program managers and providers questioned HEW commitment and motivation, “*Concerning the health extension package, they are already bored with the activity…We visit house to house and get to know some information is not true…Being bored is not about professional skill problems, they need motivation*” (project coordinator, > 5 years work experience, male). This was thoroughly refuted by the HEWs who saw themselves as custodians of community health, offering to take on additional responsibilities to safeguard the public from TB. *"We are eager to give what we know and have to the community. This is a very important service as the disease is communicable and needs attention. We are happy to serve the community"* (Parent, female, age 28).

As providers and program managers reflected upon HEW-delivered TPT, their positions about HEWs initiating TPT softened, ultimately envisioning a task-shared approach where the provider took on a supervisory role to monitor, audit and provide ongoing support to HEWs, while the HEW made home visits to initiate TPT and provide counseling. One TB focal person shared a model for supervision, *"The health extension [workers] will be supervised by the health center team in collaboration with the Kebele leaders on a weekly basis. They [should] use a checklist during supervision. They will check what has been done and what has not been done. In this way, the health center will provide support for health posts"* (TB focal person < 5 years work experience, female). For success, all stakeholder groups acknowledged training, supportive supervision and decision support tools including checklists would be needed to ensure all HEWs involved had adequate training and support to task-share TB screening, TPT initiation and TPT follow up. Moreover, materials including a registration book, referral papers, a portable scale, personal protective equipment, and means to enhance transportation for both the supervisor and the HEWs would be necessary to support the intervention.

### Theme 4: Overworked, understaffed health extension program and the need for service integration

Despite the benefits of home-based TPT, stakeholders questioned how best to implement the intervention. There were concerns about overloading an already stretched and understaffed HEW program. Each stakeholder group independently cited worker shortages as a major obstacle to HEW-initiated TPT. One provider suggested that more HEWs are needed to launch the intervention, “*There will be work overload, now you are adding another activity on the existing one, so are you changing their current load, or adding additional activity, if you add more activity, you have to add more health professionals”* (TB focal person, 5–10 years work experience, male). HEWs and providers voiced strong concerns about understaffing and their ability to meet the needs of a large rural population. Foregoing their concerns about staffing and over commitment, HEWs continued to press for the intervention, *“We may be overloaded with other tasks in the health post and the community. There are occasions in which we become reluctant or exhausted, but we should always remember that this is a matter of life-saving”* (HEW, > 10 years work experience, female).

Program managers and HEWs recognized the time required for a home visit and almost universally recommended service integration to justify the effort. Stakeholders offered differing opinions regarding which services would fit naturally with TPT but frequently suggested HIV, nutrition, and vaccination services as logical partnered services. HEWs affirmed that service integration was their standard practice and TPT fit easily within this model. One HEW explained, *“We can integrate TPT with the other home-based health care services. For instance, when I go for the home visit to offer TPT medication, I can give health education about latrine utilization, antenatal care, and screening child malnutrition, identifying vaccine defaulters, and so on. There is no reason not to integrate TPT with the other home-based health care services. An important thing, we must do it, we have to plan, set schedule and use checklist not to miss points. In this mechanism, it is possible to address all important care and services. This can simplify our activity”* (HEW, > 10 years work experience, female).

### Theme 5: Community awareness as a facilitator of TPT and of the home-based intervention

All stakeholder groups described community awareness as an essential factor in TPT success. The following sub-themes describe the far-reaching implications of community awareness and its potential impact on TPT implementation broadly and on the success of the home-based intervention.

#### Harnessing community awareness to improve community-level acceptability of home-based TPT

In response to community buy in, HEWs, providers and program managers resoundingly voiced the need for “community awareness”, a strong and emergent code. Providers perceived low TB knowledge as a barrier to optimal TPT uptake. Providers and program managers saw a need for various types of awareness of TB prevention services including pediatric screening and TPT, and of the proposed intervention to improve buy in and secure confidence by the local community. *“There is not as much a resistance if the medicine is given to them after providing the adequate information and increasing understanding about the severity of TB disease”* (Primary Health Care Unit Director < 5 years work experience, male). Healthcare education landed squarely within the HEW’s responsibilities, “*Another [HEW responsibility] is to eradicate this TB by creating awareness among our people. Our role is to educate our community, no matter where [they are], and to get free of this disease*” (HEW, > 10 years work experience, female). But stakeholders also noted the importance of utilizing existing community structures including the women’s development army, a group of female volunteers who assist HEWs by promoting health through community outreach, and local Kebele leaders.

#### Community awareness to improve TPT uptake among skeptical caregivers who view their well children as not needing medication

HEWs, providers and program managers had all encountered the sentiment that children with TB infection were “not sick” and saw this as a potential barrier to TPT acceptance and adherence, *“They say why they should take drugs without illness, children are healthy after all. My suspicion is they may raise such* *questions on why their children take drugs without, despite normal health conditions or no noticeable illness”* (HEW, > 10 years work experience, female). This concern was not specific to HEW-initiated or home-based TPT but threatened the success of the proposed intervention. A TB focal person shared, “*if you tell a family to take TPT drug, they do not accept it because they do not have the sign of illness. Unless they are informed or convinced about the issue, it is difficult. They say, ‘I am healthy, my child is healthy, there is no sign and symptom of illness what is the need for this drug*’” (TB focal person, < 5 years work experience, male) highlighting the need for better awareness around TB prevention and TPT. Across stakeholder groups, community awareness was central to improving the acceptability of TPT. Caregivers who experienced TB treatment were keenly aware of the social and psychological ramifications of inadequate knowledge about TB infection and TB disease, *“Health extension workers can provide appropriate information about this to the community to change their bad attitudes and perceptions which can improve bad feelings”* (Parent, female, age 28).

#### Community awareness to reduce stigma as a barrier to intervention acceptability

Participants at all levels of the health system were concerned that other factors like stigma would impact the acceptability of the home-based intervention. Several stakeholders discussed the frequent assumption that people living with TB also have HIV and worried that families would not want to draw unnecessary attention to their homes. Providers were concerned that home-based visits would draw negative attention to the persons being treated for TB and their household, *“The family might not be satisfied if you provide the service at home. They may perceive the service as a stigma…If you have not laid the base it would be difficult for you succeed. So you must get the families’ acceptance… Mobilizing and educating the community is a must”* (TB focal person < 5 years work experience, male). But this fear was not shared by caregivers. Many caregivers were also the person being treated for TB in their family, and while they experienced and perceived stigma and discrimination themselves as people with TB disease, they did not mention stigma related to TPT or the home-based intervention itself.

## Discussion

This qualitative study informed the design of a home-based contact investigation intervention that will be tested in a cluster-randomized trial in the East Shewa Zone of Oromia, Ethiopia. Its findings have implications for both the study and for other high TB-burden settings with trained community health workers. Participants anticipated high acceptability of the home-based intervention, feeling that it was family-centered, and that it alleviated key barriers for caregivers in the community. Stakeholders agreed that HEWs could provide TB prevention services in the community if they were task-sharing with facility-based TB focal persons and integrated into their other services to address an overworked and low staffed HEW workforce. Community awareness would be key in improving acceptability among community members, reducing stigma, and increasing caregiver acceptance of TPT for healthy children. Overall, these findings support the development of home-based contact investigation including TPT initiation and follow up. Understanding potential challenges and how they might be used to optimize home-based TB contact investigation will be critical for developing a successful intervention.

Stakeholders recognized that the home-based strategy addresses key caregiver barriers both in general and in this population [17]. While this not only includes direct gains for caregivers including saved time and money, it also improves access to TB evaluation for young children and highlights the importance of TPT by bringing it to the household level, thereby improving access to and coverage of TPT. Because home-based contact investigation addresses the people’s needs and priorities, the care model was felt to be family-centered and in line with the Stop TB Partnership’s people-centered care initiative [18]. Moreover, TPT delivery models that align with client preferences may amplify the impact of new, shorter TPT regimens [19]. Other community-based and home-based TB treatment interventions have also been found to be highly acceptable in Ethiopia and other parts of sub-Saharan Africa [6, 20, 21].

Despite this overall positive view of the intervention, there were concerns over how it would be implemented; key implementation strategies were suggested to address these concerns. The first concern was over the HEWs motivation and commitment. While providers and some program managers’ initial reactions were often skeptical, most came around to believing that HEWs could provide the services so long as they were task-shared with facility-based TB focal persons and that adequate training and supportive supervision were provided for all healthcare workers involved. The second concern regarding poor staffing and work overload could be addressed by integrating services into existing home-based services to minimize the additional workload to the health extension program. Overall, these concerns are consistent with those found in the health extension program situation analysis performed by the Ethiopian Ministry of Health as they developed their roadmap to optimize the health extension program from 2020–2035 [10]. A time and motion analysis of HEWs showed the majority of their time is spent waiting at the health post for clients to arrive and in traveling between the health post and clients’ homes [22]. The authors recommended enhancing their productivity by reducing the waiting time at the health post by increasing client demand for services and increasing time spent at the home. Providing integrated home-based TB prevention services may be one way to enhance HEW productivity. The situation analysis also realized the need to adapt to the evolving health needs of its people. Expanding the health extension program to include home-based TB contact investigation, including provision of TPT, could be one way to adapt to the evolving needs of the community while maintaining the scope and purpose of the original program. Another concern raised in the situation analysis was the HEWs knowledge and skills. As we implement the intervention, attention must be paid to adequate training of all healthcare workers involved, supportive supervision on all levels and to evaluating and understanding the accuracy of HEW TB evaluation and TPT initiation, alongside the added burden this additional work adds to a typical HEW’s workload. Overall, home-based TPT might offer an opportunity to strengthen existing HEW systems, including enhancing productivity and adapting to their evolving scope and purpose. But if home-based TPT were implemented without proper support, including training, supportive supervision and addressing the added burden, the addition of this intervention as a package of care could detract from other health-related programming.

Community awareness was a strong and emergent theme that stakeholders felt could improve acceptability of the intervention by increasing knowledge of TB prevention services in general and in relation to the planned intervention. A recent HEW assessment showed HEWs were successful in increasing awareness and improving health outcomes for a number of programs and diseases. Stakeholders also theorized that community awareness would reduce TB-related stigma. These changed perceptions of TB preventive services combined with increasing access to care were felt necessary to maximize impact of the care delivery model and short-course TPT. Recent efforts to use volunteer women groups to support TB prevention in Ethiopia, even in the absence of home-based TPT, has been successful in improving TPT uptake and completion [23]. Interestingly, despite recent surveys suggesting poor knowledge of TB in general and high levels of TB-related stigma in Ethiopia [24], the perception of stigma was stronger among healthcare providers than among clients. No caregivers brought up concerns about stigma related to TPT or TB infection. Caregivers’ fear of TB outweighed their fear of stigma, particularly among those who had experienced TB treatment. Several caregivers who experienced TB disease related stigma related to symptomatic illness and treatment but still accepted TPT for their children without mentioning concerns about isolation or status loss. This home-based TPT intervention will change the way that children and caregivers interact with the healthcare system, risking ignominy in an otherwise invisible infection. Increasing community awareness may reduce TB stigma; however, calling attention to families and social networks through home visits may also increase stigma. As we implement the intervention, attention must be paid to the unanticipated consequences of home-based treatment, both positive and negative.

### Strengths and limitations

This formative qualitative study, embedded in a larger clinical trial, ensured rigor by establishing credibility through triangulation across stakeholder groups and established confirmability through audit trails and analyst reflexivity. Through this research, we were able to understand and be responsive to the needs of program managers, providers, HEWs and caregivers to develop context-specific messaging and implementation strategies to enhance acceptability of home-based TB prevention services. Key modifications that resulted from this work included adding a task-shared approach to TPT initiation and an enhanced community-engagement plan. These context-specific adaptations will likely increase the acceptability of the intervention in the CHIP-TB trial. While this study was successfully built into a larger cluster-randomized implementation effectiveness trial, some challenges specific to conducting the qualitative research as part of a larger trial included ensuring adequate time for data collection and analysis prior to trial initiation and provision of adequate resources to oversee both research teams.

The cross-sectional nature of this study did not allow us to explore how these complex and dynamic processes may work and change over time. Triangulating across a wide variety of stakeholder groups allowed for nuanced insights into the potential strengths and limitations of the home-based approach. Though key stakeholder groups were well represented in the research, pastoralist and urban communities were not engaged. In the past, inadequate contextualization of the health extension program to these communities hindered implementation of these services [10]. Others have found that community-based and home-based approaches in these settings often require further modification and consideration due to their separate structural organization to improve acceptability [25]. Additional work may be needed to adapt this intervention to those communities.

## Conclusion

Our study showed that home-based contact management with TPT initiation and follow up of close child contacts less than 15 years of age was an acceptable strategy for program managers, TB focal persons, HEWs and caregivers, but that the intervention should be task-shared between HEWs and TB focal persons and be integrated into other household activities to reduce the burden on HEWs. Moreover, an active community engagement strategy should be developed to support community awareness to improve TPT uptake, particularly among those skeptical of providing medication to their otherwise healthy children and reduce stigma. While awaiting the findings of the CHIP-TB study, these formative results can help guide national and district-level policy by emphasizing the acceptability of a family-centered, home-based strategy for TPT services for child contacts of persons with TB in Ethiopia and other similar settings.

## Supplementary Information

Supplementary Material 1.

Supplementary Material 2.

## Data Availability

De-identified textual data can be made available upon reasonable request to the corresponding author.

## Abbreviations

HEW: Health extension worker
TB: Tuberculosis
TPT: Tuberculosis preventive treatment
3HP: 3 Months of weekly rifapentine and isoniazid
3RH: 3 Months of daily rifampicin and isoniazid
COVID-19: Coronavirus Disease of 2019

## References

[1] Global tuberculosis report 2023. Geneva: World Health Organization; 2023. Licence: CC BY-NC-SA 3.0 IGO.

[2] Roadmap towards ending TB in children and adolescents teGWHOLCB-N-SI.

[3] Suthar AB, Ford N, Bachanas PJ, et al. Towards universal voluntary HIV testing and counselling: a systematic review and meta-analysis of community-based approaches. PLoS Med. 2013;10(8):e1001496. 10.1371/journal.pmed.1001496. 23966838 10.1371/journal.pmed.1001496 PMC3742447

[4] Nachega JB, Adetokunboh O, Uthman OA, et al. Community-based interventions to improve and sustain antiretroviral therapy adherence, retention in HIV care and clinical outcomes in low- and middle-income countries for achieving the UNAIDS 90–90-90 targets. Curr HIV/AIDS Rep. 2016;13(5):241–55. 10.1007/s11904-016-0325-9. 27475643 10.1007/s11904-016-0325-9 PMC5357578

[5] Arshad A, Salam RA, Lassi ZS, Das JK, Naqvi I, Bhutta ZA. Community based interventions for the prevention and control of tuberculosis. Infect Dis Poverty. 2014;3:27. 10.1186/2049-9957-3-27. 25136445 10.1186/2049-9957-3-27 PMC4136404

[6] Bonnet M, Vasiliu A, Tchounga BK, et al. Effectiveness of a community-based approach for the investigation and management of children with household tuberculosis contact in Cameroon and Uganda: a cluster-randomised trial. Lancet Glob Health. 2023;11(12):e1911–21. 10.1016/S2214-109X(23)00430-8. 37918417 10.1016/S2214-109X(23)00430-8

[7] Ahmed S, Kim MH, Dave AC, et al. Improved identification and enrolment into care of HIV-exposed and -infected infants and children following a community health worker intervention in Lilongwe. Malawi J Int AIDS Soc. 2015;18:19305. 10.7448/IAS.18.1.19305. 25571857 10.7448/IAS.18.1.19305 PMC4287633

[8] Malhotra A, Nonyane BAS, Shirey E, et al. Pragmatic cluster-randomized trial of home-based preventive treatment for TB in Ethiopia and South Africa (CHIP-TB). Trials. 2023;24(1):475. 10.1186/s13063-023-07514-7. 37491264 10.1186/s13063-023-07514-7 PMC10367260

[9] Federal Democratic Republic of Ethiopia Ministry of Health. National Guidelines for TB, DR-TB and Leprosy in Ethiopia. Addis Ababa, Ethiopia: 2018.

[10] Ethiopia Ministry of Health. Realizing Universal Health Coverage through Primary Healthcare: A Roadmap for Optimizing the Ethiopian Health Extension Program 2020–2035. Addis Ababa, Ethiopia: 2020.

[11] Coffey P, Sharma J, Gargi K, Neupane D, Dawson P, Pradhan Y. Feasiblity and Acceptability of gentamicin in the Uniject prefilled injection system for community-based treatment of possible neonatal sepsis: the experience of female community health volunteers in Nepal. J Perinatol. 2012;32:959–65. 22422117 10.1038/jp.2012.20

[12] Sazawal S, Black RE, Pneumonia Case Management Trials G. Effect of pneumonia case management on mortality in neonates, infants, and preschool children: a meta-analysis of community-based trials. Lancet Infect Dis 2003;3(9):547–56. (https://www.ncbi.nlm.nih.gov/pubmed/12954560). 10.1016/s1473-3099(03)00737-0 12954560

[13] Baqui AH, Arifeen SE, Williams EK, et al. Effectiveness of home-based management of newborn infections by community health workers in rural Bangladesh. Pediatr Infect Dis J. 2009;28(4):304–10. 10.1097/INF.0b013e31819069e8. 19289979 10.1097/INF.0b013e31819069e8 PMC2929171

[14] Kidane G, Morrow RH. Teaching mothers to provide home treatment of malaria in Tigray, Ethiopia: a randomised trial. Lancet. 2000;356(9229):550–5. 10.1016/S0140-6736(00)02580-0. 10950232 10.1016/S0140-6736(00)02580-0

[15] Assefa Y, Gelaw YA, Hill PS, Taye BW, Van Damme W. Community health extension program of Ethiopia, 2003–2018: successes and challenges toward universal coverage for primary healthcare services. Global Health. 2019;15(1):24. 10.1186/s12992-019-0470-1. 30914055 10.1186/s12992-019-0470-1 PMC6434624

[16] ATLAS.ti Scientific Software Development GmbH. (2021). ATLAS.ti (version 9.1.7.0) [Qualitative data analysis software]. https://atlasti.com.

[17] Szkwarko D, Hirsch-Moverman Y, Du Plessis L, Du Preez K, Carr C, Mandalakas AM. Child contact management in high tuberculosis burden countries: A mixed-methods systematic review. PLoS ONE. 2017;12(8):e0182185. 10.1371/journal.pone.0182185. 28763500 10.1371/journal.pone.0182185 PMC5538653

[18] The Stop TB Partnership. The RTC Toolkit: People-centered design for the optimal, rapid adn sustainable roll-out of TB innovations. Re-imagining TB Care. Geneva, Switzerland: 2023. https://www.stoptb.org/people-centered-design-toolkit.

[19] Strauss M, Wademan DT, McInziba A, et al. TB preventive therapy preferences among children and adolescents. Int J Tuberc Lung Dis. 2023;27(7):520–9. 10.5588/ijtld.22.0645. 37353873 10.5588/ijtld.22.0645 PMC10321362

[20] Tulloch O, Theobald S, Morishita F, et al. Patient and community experiences of tuberculosis diagnosis and care within a community-based intervention in Ethiopia: a qualitative study. BMC Public Health. 2015;15:187. 10.1186/s12889-015-1523-x. 25885789 10.1186/s12889-015-1523-x PMC4349713

[21] Hirsch-Moverman Y, Howard AA, Yuengling KA, et al. Effectiveness of a community-based intervention to prevent childhood TB in Lesotho. Int J Tuberc Lung Dis. 2022;26(7):612–22. 10.5588/ijtld.21.0558. 35768915 10.5588/ijtld.21.0558

[22] Tilahun H, Fekadu B, Abdisa H, et al. Ethiopia’s health extension workers use of work time on duty: time and motion study. Health Policy Plan. 2017;32(3):320–8. 10.1093/heapol/czw129. 27658649 10.1093/heapol/czw129

[23] Jerene D, Assefa D, Tesfaye K, et al. Effectiveness of women-led community interventions in improving tuberculosis preventive treatment in children: results from a comparative, before-after study in Ethiopia. BMJ Open. 2022;12(7):e062298. 10.1136/bmjopen-2022-062298. 35863840 10.1136/bmjopen-2022-062298 PMC9310159

[24] Datiko DG, Jerene D, Suarez P. Stigma matters in ending tuberculosis: Nationwide survey of stigma in Ethiopia. BMC Public Health. 2020;20(1):190. 10.1186/s12889-019-7915-6. 32028914 10.1186/s12889-019-7915-6 PMC7006204

[25] Megerso A, Deyessa N, Jarso G, Tezera R, Worku A. Exploring community tuberculosis program in the pastoralist setting of Ethiopia: a qualitative study of community health workers’ perspectives in Borena Zone, Oromia Region. BMC Health Serv Res. 2021;21(1):632. 10.1186/s12913-021-06683-y. 34210297 10.1186/s12913-021-06683-y PMC8252211

