# Supplementary material for: Improving access to tuberculosis preventive treatment for children in Ethiopia: designing a home-based contact management intervention for the CHIP-TB trial through formative research
Source: BMC Health Serv Res. 2024 Sep 10;24:1043. doi: 10.1186/s12913-024-11451-9 (PMC11386379; doi:10.1186/s12913-024-11451-9)
Supplement: Supplementary file 1 — Supplementary Material 1. [file 12913_2024_11451_MOESM1_ESM.pdf]

PIN: ETH ------- __ __

Protocol: A Qualitative Study to Optimize a Community-Based Pediatric TB/HIV Prevention Intervention and Implementation Strategy

Key Informant Interview Guide (Providers/Program Managers/Policy Makers)

City: ____

Clinic Name: ____

Date of interview: □□/□□/□□□□ **(DD/MM/YYYY)** **Time:** □□:□□

INTRODUCTION:

Thank you for speaking to me today. I am interested in your views and experiences related to your involvement with pediatric TB services for people living with HIV in Ethiopia, and your thoughts on how to improve these services. We appreciate you taking the time to talk with us. There are no right or wrong answers. I am here to learn from you. You may stop the interview at any time and you may choose to not answer any question you would prefer not to answer.

Do you have any questions before we start?

Background information:

First, I would like to start by asking you some questions about your background and your role.

[Please write information in or circle appropriate response]

D1. Participant Gender: **Male** **Female** (circle)

D2: Participant Training: (circle)

Doctor	Nurse	Community Health Worker
Public Health		Other: ______________________

D3. Participant Job Title: __________________________

D4. For program managers/policy makers: How long have you been working in this role?
____

D5. For clinic staff: How long have you worked in this clinic? ___________________

Open-ended questions:

Let's talk about your job and your role in TB prevention among household child contacts

1. Please tell me a bit about your job and your role in the health care system.

   Probe: What are your duties?
   Do you have any other duties?

2. What is your role and the role of your department in tackling TB prevention among household contacts?

   Probe: Tell me more about that part of your job…
   Probe: How big a portion of your job is TB services?

3. Tell me about the process of identifying children who live in the household of a TB index patient and linking them to care.

   Probe: Which staff are involved in listing or confirming the household contacts of TB patients?
   Does this process occur in the clinic or in the home?
   Once children are identified, who makes sure they are evaluated in clinic? How is that link to care accomplished?
   How different is this process for each index patient?

4. How do you feel the current process of identifying children living in the household of a TB index patient is working?
   Probe: What is working well for identifying household child contacts?
   What isn't working as well?
   What could be improved?

5. How do you feel the current process for linking household child contacts to TB prevention services is working?
   Probe: What is working well about linking household child contacts to care?
   What isn't working as well?
   What could be improved?

Research has shown at least half of children who live in the home of a TB index patient never come to the clinic to be screened for TB and therefore do not start TB preventive therapy or TB treatment. We will be studying a home-based intervention to identify, evaluate and initiate TB preventive therapy in children in the home. Let's talk about how a community-based intervention for TB prevention can best be structured to be successful.

6. If you were tasked with coming up with a home-based intervention for TB prevention, what would you recommend or implement?
   Probe: What types of health care workers should go to the house?
   How often should home visits be made?

How can this be combined with other home-based services already established in Ethiopia?
Who should initiate TPT?

7. What do you think are the benefits of evaluating children at home and initiating TB preventive therapy there? Please explain.

   Probe: How will families feel about having their children evaluated in the home?
   How will families feel about not having to visit the clinic?

8. From your perspective as a [job title], what challenges do you foresee in doing home-based evaluation and initiation of TB preventive therapy? Please explain.
   Probe: What will be the challenges for the TB program?
   What will be the challenges for the clinic TB nurse?
   What will be the challenges for the health extension worker?
   What will be the challenges for the health extension worker?
   What will be the challenges for the family?
   What do you think is the best way to reach school-aged children?

9. How can some of these challenges be addressed?
   Probe: Ask challenge-by-challenge

10. Should this type of home-based intervention be packaged with other health services? If yes, what services do you recommend being offered at the home in addition to TB services like contact tracing and DOT for index patients?
    Probe: What about HIV testing and/or PMTCT?
    What about nutrition or immunization programs?
    Other programs?

11. What challenges do you foresee in combining this intervention with other home-based services? How might we solve these challenges?

12. Please list all the different things you can think of that are needed to make a home-based intervention work well in Ethiopia.

    Probe: What else comes to mind? Repeat/review list. Anything else?

13. Now you just listed some strategies of things you think are needed to make a home-based intervention work well in Ethiopia. Now I would like you to rank those things you just mentioned in order of importance from one through three. One is the most important and three is less important. (if there are only two strategies listed, can have participant rank them 1 and 2).

    Note to interviewer: After the participant has ranked the strategies from one to three, repeat the order of strategies to the participant to confirm and to ask if they have any additional details to add.

Let's talk about program implementation for a home-based intervention for child contact evaluation and TPT initiation. In other words, how the intervention can be supported to produce successful clinical outcomes in Ethiopia

14. How are the community-based care teams structured and supervised?
    Probe: How many HCW (by type) per team? How many teams per clinic?
    How are these teams supervised?
    How are disagreements handled between programs and home-based care teams?
    What types of transportation / funding are provided to community health team?

15. How do you think the TB and HIV services provided by health extension workers should be supervised?
    Probe: Who should the home-based teams be responsible to? TB nurse? home-based care team supervisor? Clinic manager?

16. What types of clinical support tools are most helpful to assist health extension workers in communicating with patients? Why?
    Decision support tools built into patient records
    Provider checklists
    Posters
    Job aids

17. **Program Manager and Provider:**
    In your opinion, what's the best way for health extension workers to communicate to the TB nurse/program about TB services they provided?

    How is this currently done?
    What other suggestions do you have to improve this communication?

    or
    Health Extension Worker:
    In your opinion, what's the best way for you to communicate to the TB nurse/program about TB services you provide in the community?
    How is this currently done?
    What other suggestions do you have to improve this communication?

18. **Program Manager or Provider**:
    If a health extension worker identifies a problem with the way the system works here at the clinic or at the health post, would they feel empowered to communicate their concerns to a clinic nurse or direct manager?
    Probe: How do your peers (and/or you) react to criticism or to challenges reported to you by those under your management?
    or
    Health Extension Worker:

If you identify a problem with the way the system works here at the clinic or at the health post, what would you do to address the problem?

Probe: Who would you report to about the problem identified?
How would your feedback be received?
- Have you ever experienced this? How was the feedback received? What was the outcome ?

19. **Program Manager or Provider:**
Can you describe a time where you gave feedback to a health extension worker?
Probe: What was good about the way the feedback was given?
How could it have been improved?
Did you find the feedback effective? Did you make any changes?
or
Health Extension Worker:
Can you describe a time where you received feedback?
Probe: What was good about the way the feedback was given?
How could it have been improved?
Did you find the feedback effective? Did you make any changes?

20. **Program Manager or Provider:**
In your opinion, among health extension workers, how much learning occurs from peers and experiences versus training from superiors?
Probe: How important are each type of learning to a health extension worker?
or
Health Extension Worker:
In your opinion, how much learning occurs between you and other health extension workers versus training from superiors?

21. When you think about the most successful trainings you've been to, what has made those trainings successful?
What did you like about that training? What didn't you like about that training?
What about these trainings has made you feel comfortable to make changes in your practice?
If you haven't been to a successful training, how would you change the trainings you have attended to improve them?
How often do clinic nurses/doctors/HEWs receive training on TB? When was the last training? Who provided the training?

22. If you had to design an ongoing mentorship program to assist health extension workers in providing home-based TB prevention services for child TB contacts, what would it look like? (ongoing mentorship is mentorship provided over time after an initial training has taken place)
How often would health extension workers receive mentorship and feedback?

23. In addition to training physicians, nurses and community health teams on their respective roles in contact training, TB screening, diagnosis and treatment or prevention, what other training will be needed to make this intervention successful?

Is there anything else you would like to share with me?

Thank you for your time. We appreciate all your inputs and suggestions.

End time: ☐☐:☐☐
