# Supplementary material for: Improving access to tuberculosis preventive treatment for children in Ethiopia: designing a home-based contact management intervention for the CHIP-TB trial through formative research
Source: BMC Health Serv Res. 2024 Sep 10;24:1043. doi: 10.1186/s12913-024-11451-9 (PMC11386379; doi:10.1186/s12913-024-11451-9)
Supplement: Supplementary file 2 — Supplementary Material 2. [file 12913_2024_11451_MOESM2_ESM.pdf]

PIN: ETH-_____- _____

Protocol: A Qualitative Study to Optimize a Community-Based Pediatric TB/HIV Prevention Intervention and Implementation Strategy

In-Depth Interview Guide (Child Caregivers)

Eligibility:

Caregiver of a tuberculosis-exposed child in the last 12 months

City: ____

Clinic Name: ____

Date of interview: □□/□□/□□□□ **(DD/MM/YYYY)** **Time:** □□:□□

INTRODUCTION:

Thank you for speaking with me today. As we discussed I am interested in your experiences related to the provision of TB prevention services here in this clinic, and your thoughts on new ways to improve these services in the future. We appreciate you taking the time to talk with us. There are no right or wrong answers. I am here to learn from you. You may stop the interview at any time and you may choose to not answer any question you would prefer not to answer.

Do you have any questions before we start?

Background information:
First, I would like to start with gathering some information about you and your background.

[Please write information in or circle appropriate response]

D1. Participant Gender: Male Female (circle)

D2. What is your age? ___________________

D3. What is your highest level of education? ______________

D4. What is your occupation? ____

D5. How many children under the age of 5 are you the primary caregiver for in your household? ______

D6. How many children between the ages of 5 and 15 are you the primary caregiver for in your household? _______

D7. What is your relationship to these children? (mark all that apply)
Parent Grandparent Aunt/Uncle Other

D8. Who in the household had TB?
Respondent Other Parent Child Other household member

Open-ended questions:

I would like to hear a bit about your and your child's experience with the clinic in general.

1. Please describe the different types of treatment your child got at the local clinic

2. How do you feel about the services your child got at the clinic?

I would like to hear a bit about your and your child's experience with health extension workers in general.

3. What type of health services has your child received in the home or at the health post, if any?

4. How do you feel about the services your child got at home or at the health post?
   Probe: What do you like?
   What do you dislike?
   What could be improved?

5. In what ways are health extension workers helpful in general?
   Probe: How have you seen them be helpful to your community overall?

6. Is there anything you would change about how health extension workers work with the community?
   Probe: Tell me more about what you would like to see these workers do differently or in addition to what they currently do?

Now I would like to talk a bit more specifically about any experiences your child has had with TB services at the clinic

7. How do you feel about the services your child got for TB at the clinic?
   Probe: What did you like? What did you dislike? What could be improved?

8. I understand your child(ren) was(were) given a medicine to prevent TB. This may have been called isoniazid or may have been two medications called either rifampicin and isoniazid or rifapentine and isoniazid (3HP). Tell me about you and your child's experience with the clinic receiving and using this medication.

Note to interviewers: This medication was most likely isoniazid or "IPT", but in a small number of patients in one clinic, they may also have received two medications called rifapentine and isoniazid or 3HP once weekly for three months.

Probe: How does a caregiver find out their child has been exposed to TB?
How does a caregiver find out their child needs to be evaluated for TB?

How does a caregiver know when their child should be treated with TB preventive therapy?
Did your child undergo any procedures or testing?
How did your child get started on TB preventive treatment?

9. Tell me about your experience with refilling that medication in the clinic, if any?
Probe: What challenges did you have in getting those refills?

10. While you were getting or refilling the medication, what do you think the clinic did well?
Probe: What can the clinic do well and should continue to do in the future?

11. While you were getting or refilling the medication, what do you think the clinic could have done better?
Probe: What can the clinic change to help you and other families in the future?

Now I'd like to talk about your feelings on how the clinic may be able to provide care to prevent TB in the home.

12. To protect children from developing TB a medicine is provided called isoniazid to prevent TB. OR If your child were to need this medication, how would you prefer your child to receive treatment/care?
Probe: Would you prefer home visits or clinic visits?

13. What are the reasons for you preferring home visits or clinic visits?

14. How do you feel about a health extension worker visiting your home for the purposes of TB prevention care for your child?

15. How would you feel about a health extension worker starting your child on medication to prevent TB in your home?

Note to interviewers: can use name of specific TB medication family used, if known. e.g. isoniazid, rifampin/isoniazid, rifapentine/isoniazid (3HP).

Probe: What are the advantages?
What are the disadvantages?
Is it the same for young children versus older children/adolescents?

16. How would you feel about a health care worker delivering refills of medication to prevent TB to your child in the home?

17. How do you feel about testing your child for HIV in the home?

Note to interviewer: If asked, HIV testing would be a rapid (e.g. a finger prick test). Testing for infants < 1 year ("DBS" or dried blood spot) would need to be done in the clinic.

Probe: What are the advantages?
Probe: What are the disadvantages?
Probe: Any differences in opinion for younger children versus older children?

18. What other services or activities would you like a health care worker to help you with regarding health care for your child in the home?

19. Are there any other ideas that you may have about how the health care system could improve TB prevention services for the caregivers or for their children?

20. Tell me all the things the clinic and health extension workers need to do to make sure children get onto medicine to prevent TB

Probe: What else comes to mind? Repeat/review list. Anything else?

21. Please rank the top three ways you think the clinic and health extension workers can make sure children get onto medication to prevent TB.

Note to interviewer: After the participant has ranked the strategies from one to three, repeat the order of strategies to the participant to confirm and to ask if they have any additional details to add

Is there anything else you would like to share with me?

Thank you for your time. We appreciate all your inputs and suggestions.

End time: ☐☐:☐☐
